# Supplementary material for: 3D-Printed Phenylboronic Acid-Bearing Hydrogels for Glucose-Triggered Drug Release
Source: Polymers (Basel). 2024 Sep 3;16(17):2502. doi: 10.3390/polym16172502 (PMC11398034; doi:10.3390/polym16172502)
Supplement: Supplementary file 1 [file polymers-16-02502-s001.zip › polymers-3141677-supplementary.pdf]

Supporting Information

3D Printed Phenylboronic Acid-bearing Hydrogels for Glucose-Triggered Drug Release

Jérémy Odent*, Nicolas Baleine, Serena Maria Torcasio, Sarah Gautier, Olivier Coulembier, Jean-Marie Raquez

Laboratory of Polymeric and Composite Materials (LPCM), Center of Innovation and Research in Materials and Polymers (CIRMAP), University of Mons (UMONS), Place du Parc 20, 7000 Mons, Belgium

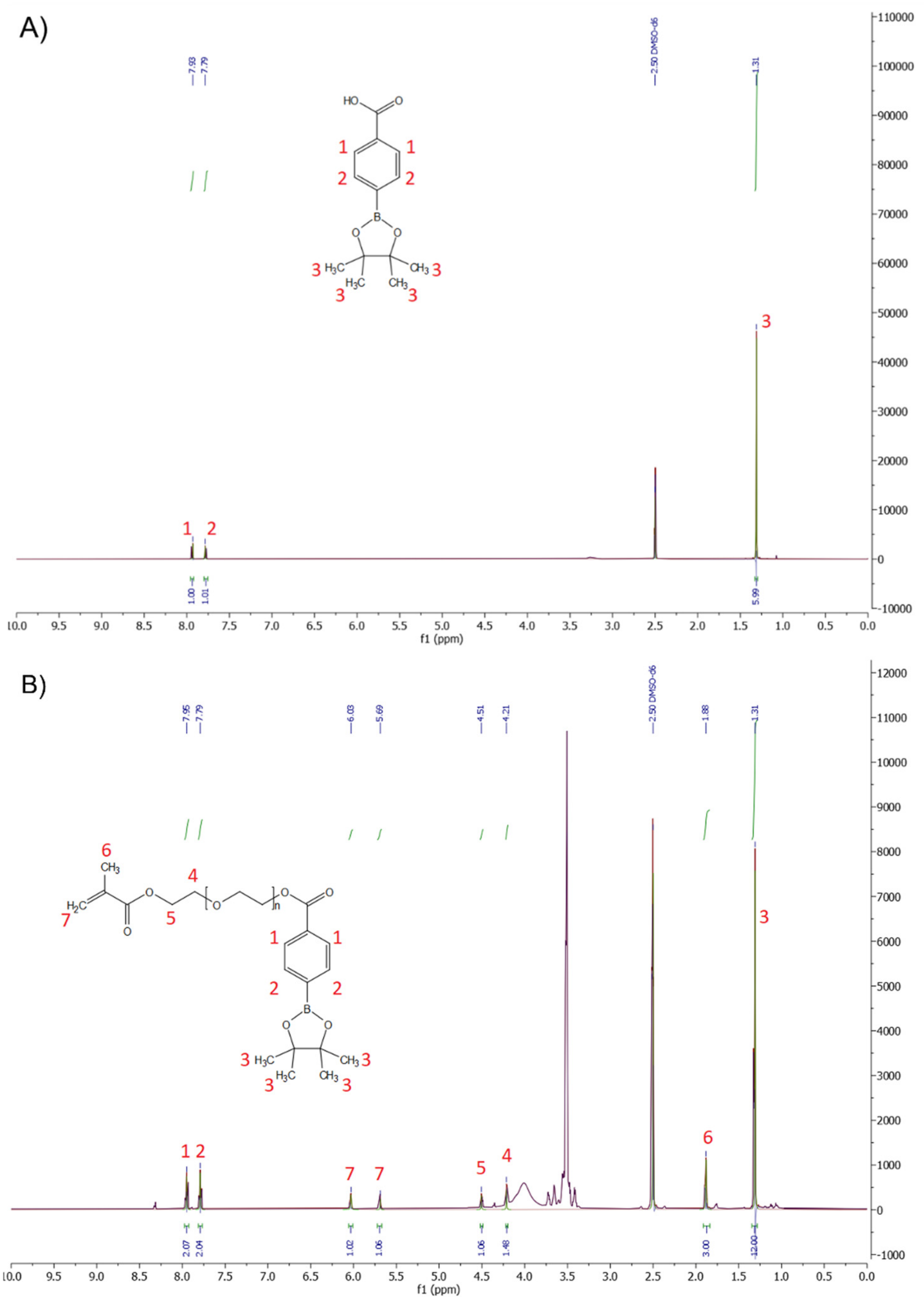

Figure S1. ^{1}H-NMR in DMSO-d_6 of (A) pinacol-protected phenylboronic acid and (B) PEGMA-PBA-Pinacol. The acid proton resonance around 12 ppm is not shown.

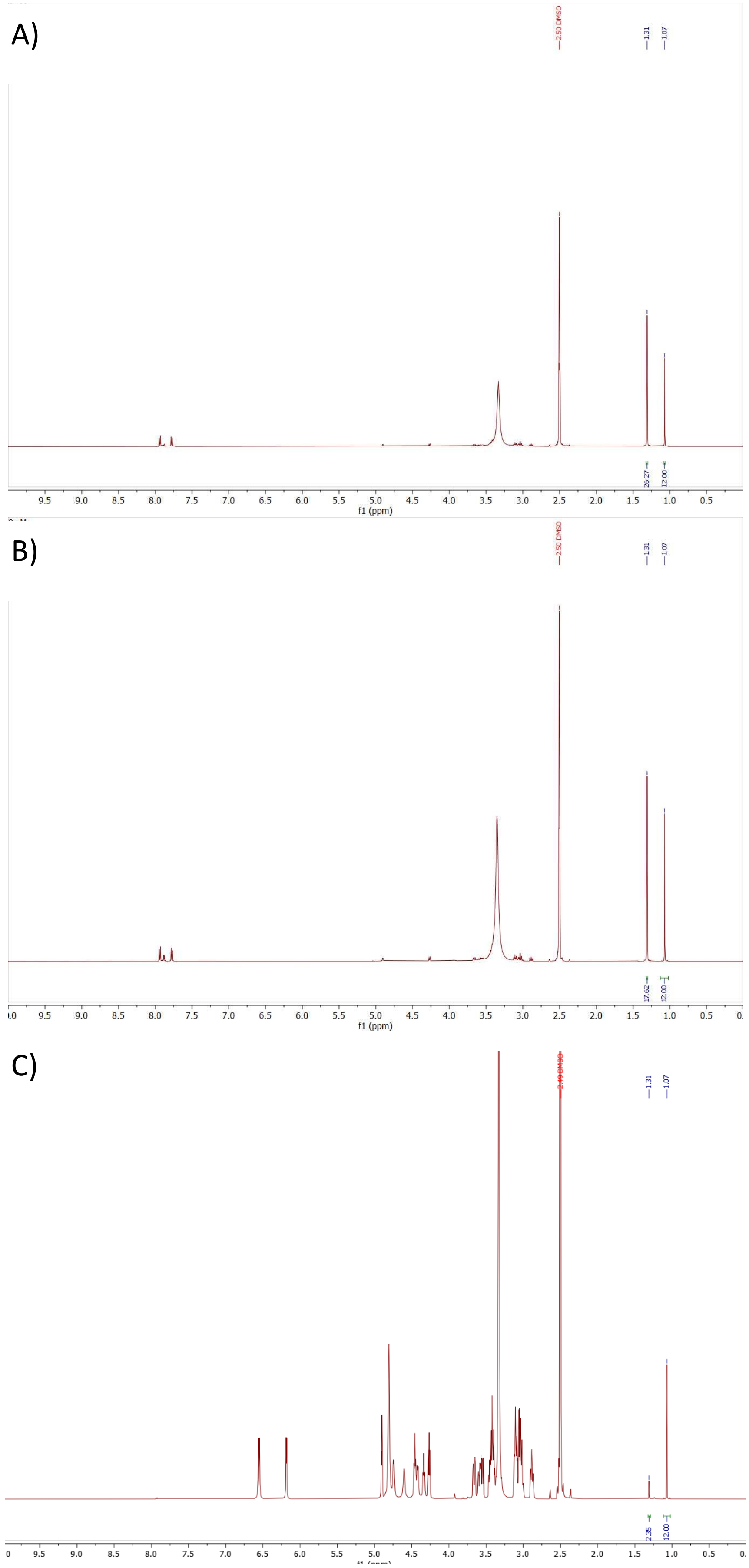

Figure S2. ^{1}H-NMR in DMSO-d_6 of the crude medium obtained after 3 days of reaction between PEGMA-PBA-Pinacol in a A) 4 mM, B) 8 mM, and C) 12 mM glucose solution (using a 100 mM sodium phosphate buffer).

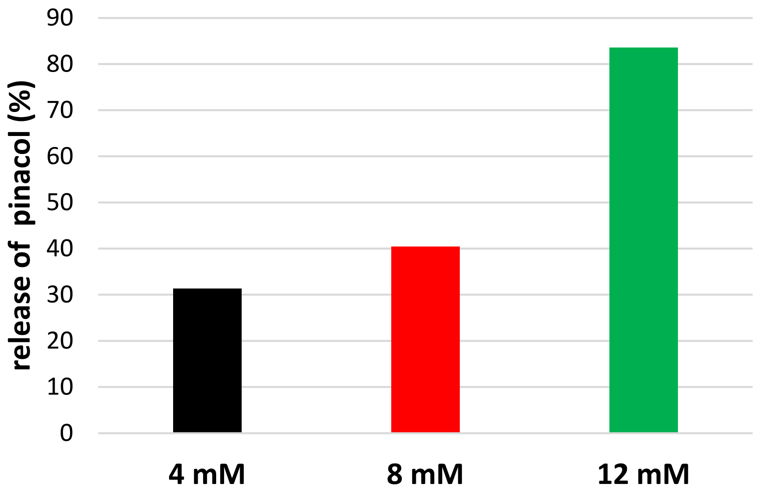

Figure S3. Release of pinacol from PEGMA-PBA-Pinacol after 3 days in a 4 mM, 8 mM, and 12 mM glucose solution (as-determined by ^{1}H-NMR).

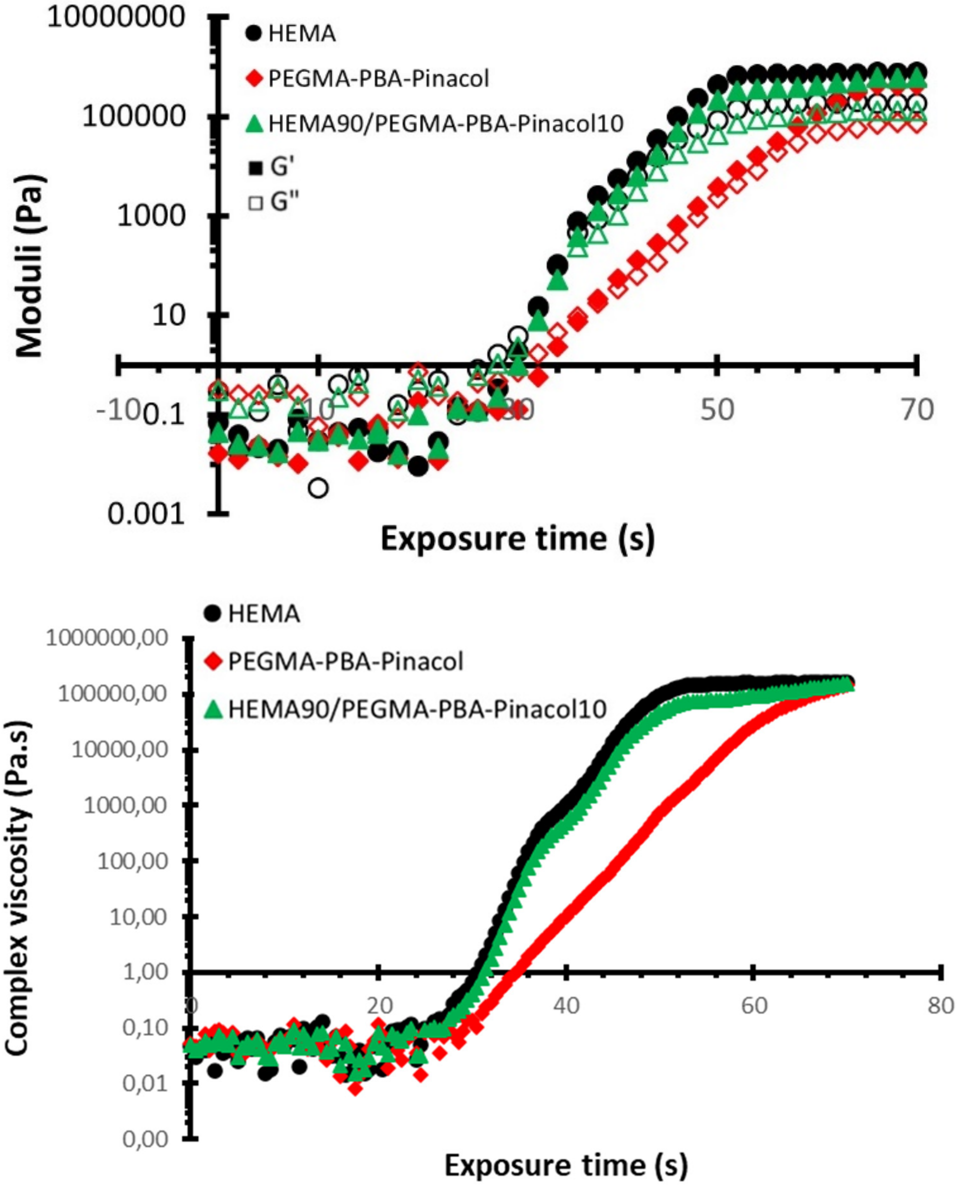

Figure S4. Storage modulus G' (full) and loss modulus G'' (hollow) (A) as well as complex viscosity (B) of HEMA (black circle), PEGMA-PBA-pinacol (red diamond) and HEMA/PEGMA-PBA-pinacol (90/10 mol%) (green triangles) as a function of near-UV exposition time.

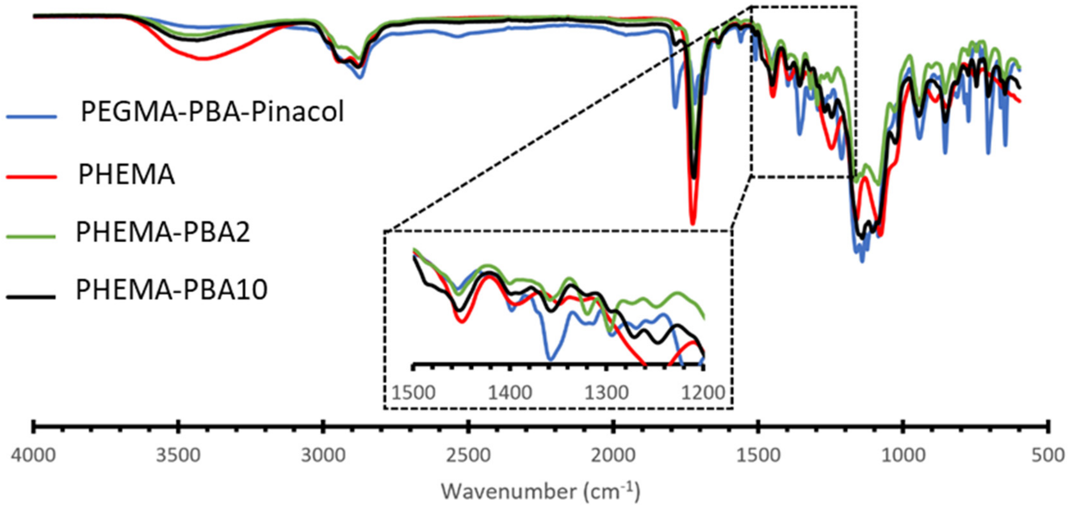

Figure S5. FTIR spectra of PEGMA-PBA-Pinacol (blue), neat PHEMA (red), PHEMA-PBA2 (green) and PHEMA-PBA10 (zoom in the region of borate asymmetric stretching vibration around 1350 cm^{-1}).

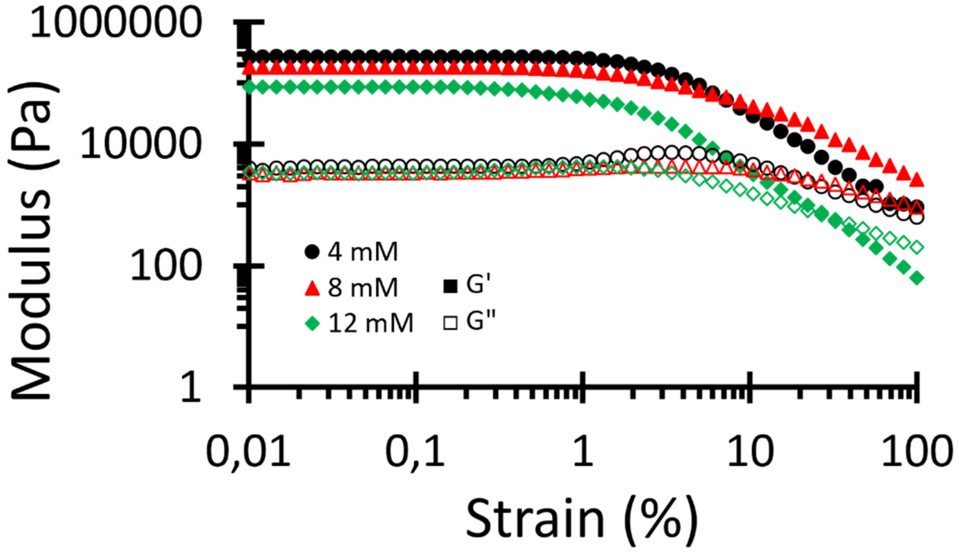

Figure S6. Storage modulus G’ (full) and loss modulus G” (hollow) as a function of strain of PHEMA-PBA10 swelled at equilibrium in glucose solutions of 4 mM (black circles), 8 mM (red triangles) and 12 mM (green diamonds).

Table S1. Rheological characterization and associated mesh sizes of PHEMA-based hydrogels.

Hydrogel composition	Glucose concentration (mM)	G' (kPa)	r_{mesh} (nm)*
Neat PHEMA	0	41	5.7
	4	41	5.7
	8	41	5.7
	12	41	5.7
PHEMA-PBA2	0	39	5.8
	4	272	3.1
	8	187	3.5
	12	55	5.2
PHEMA-PBA5	0	38	5.8
	4	357	2.8
	8	233	3.3
	12	59	5.1
PHEMA-PBA10	0	37	5.9
	4	470	2.5
	8	285	3.0
	12	66	4.9

$$^{*}r_{mesh} = \sqrt[3]{\frac{6RT}{\pi N_{Av} G'}}$$

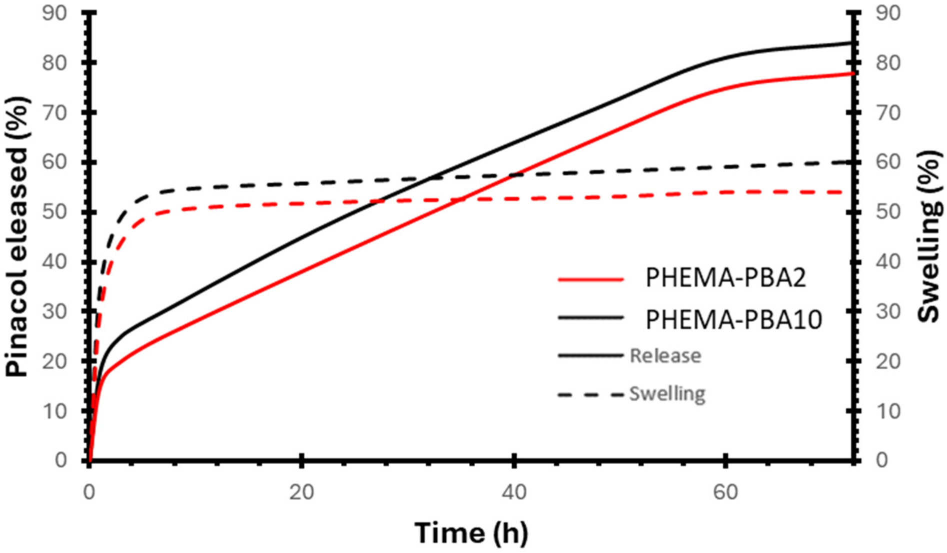

Figure S7. Swelling behavior (dashed line) and release kinetic (solid line) of PHEMA-PBA2 (red) and PHEMA-PBA10 (black) in a glucose solution of 12 mM.

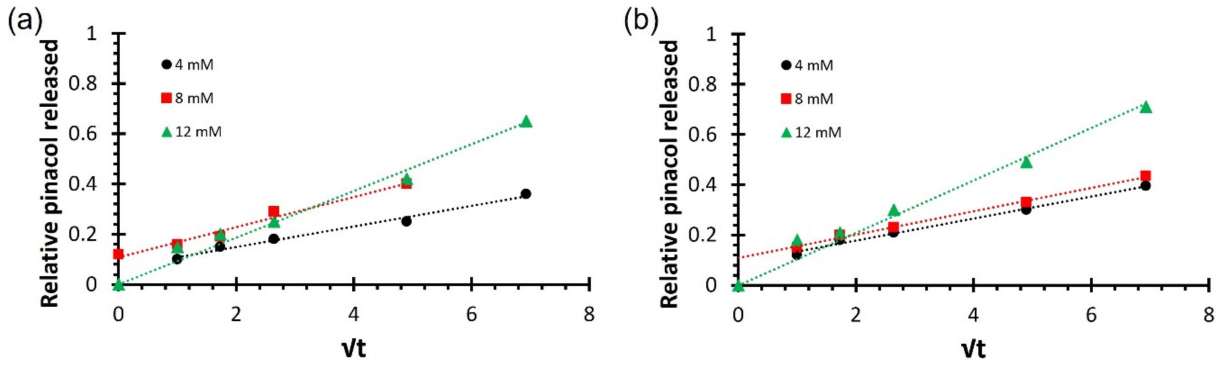

Figure S8. Relative pinacol released as a function of square root of time for diffusion coefficient determination of (a) PHEMA-PBA2 and (b) PHEMA-PBA10.

Table S2. Diffusion coefficient D (cm²/s) of pinacol release from PHEMA-based hydrogels.

Composition	Glucose solution (mM)	Diffusion coefficient D (cm²/s)
PHEMA-PBA2	4	$3.12\ 10^{-12}$
	8	$6.66\ 10^{-12}$
	12	$1.61\ 10^{-11}$
PHEMA-PBA10	4	$3.59\ 10^{-12}$
	8	$4.01\ 10^{-12}$
	12	$2.02\ 10^{-11}$

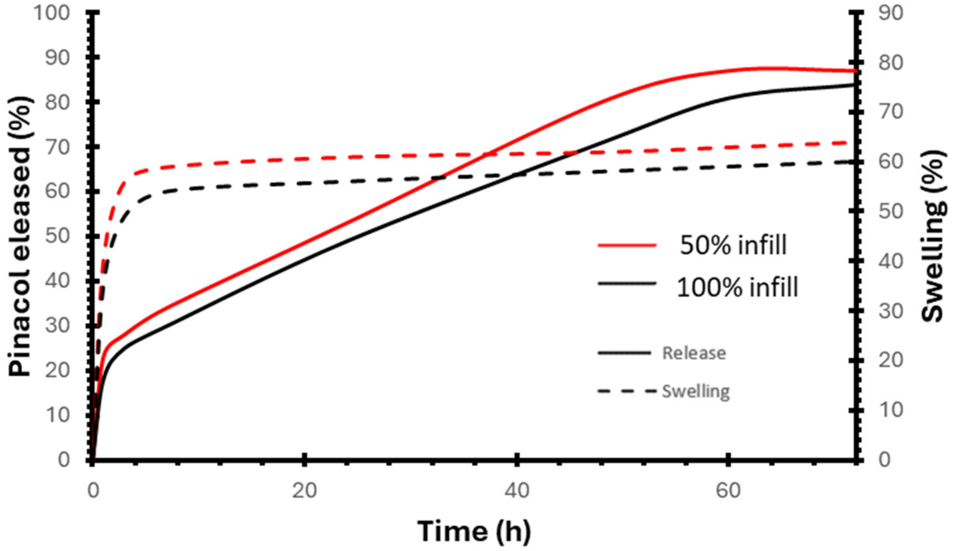

Figure S9. Swelling behavior (dashed line) and release kinetic (solid line) of implant with an infill density of 100% (black) and 50% (red) in a glucose solution of 12 mM.
